# Prioritizing the protection of welfare in gene-edited livestock

**DOI:** 10.1093/af/vfz053

**Published:** 2020-01-10

**Authors:** Adam Shriver

**Affiliations:** 1 Wellcome Centre for Ethics and Humanities, University of Oxford, Oxford, UK; 2 Oxford Uehiro Centre for Practical Ethics, University of Oxford, Oxford, UK

**Keywords:** animal welfare, bioethics, CRISPR, gene editing, genetic engineering

**Implications**

- We should adopt a principle for the conservation of welfare to ensure that the genetic modification of livestock does not result in unnecessary suffering.
- Failing to do so would both be morally wrong and likely to result in a serious undermining of public trust in food producers.
- This principle needs to be enshrined in legislation or regulation to be effective.

## Introduction

In 1995, the philosopher Bernard Rollin proposed what he called the principle for the conservation of welfare. This principle stated that, “any animals that are genetically engineered for human use should be no worse off, in terms of suffering, after the new traits are introduced into the genome than the parent stock was prior to the insertion of the new genetic material” (Rollin, 1995, p. 169). In what follows, I will argue that the global community ought to adopt a modified version of this principle in regard to all genetic modification of animals performed for nonresearch purposes. Moreover, I suggest that the principle should be enshrined in the law or regulations, and that assuming that the principle will be followed via “self-regulation” would be both morally wrong and likely to permanently damage trust in food producers.

## Defining and Defending the Principle for the Conservation of Welfare

**Figure 1.**
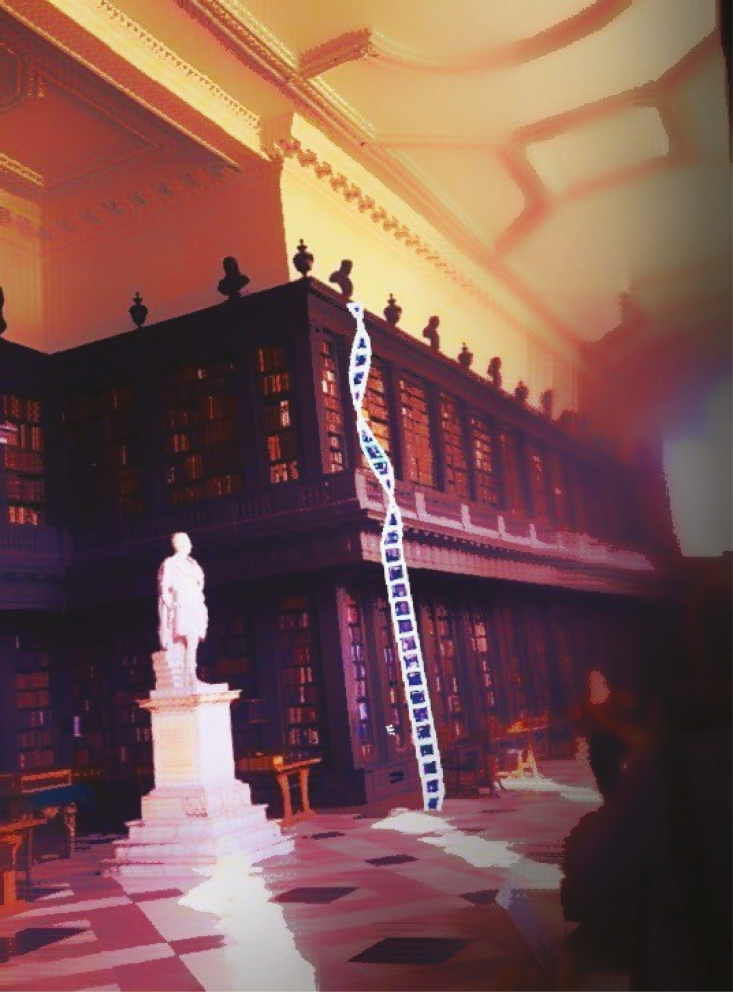
Gene editing is rapidly transforming our knowledge of biology. Image credit: Laurie Shriver.

**Figure 2.**
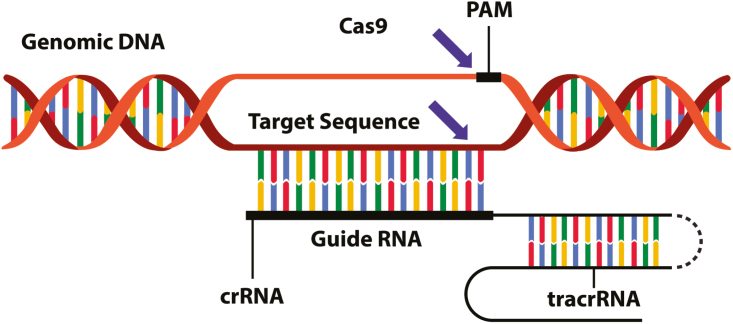
New gene editing technologies allow precise modification of the genetic code without insertion of new genetic material.

Our ability to alter the genetic code of plants and animals has become far more precise and efficient since the time Rollin first proposed the principle for the conservation of welfare, and the definition should be changed accordingly (Figure 1). Techniques such as transcription activator-like effector nucleases, and clustered regularly interspaced short palindromic repeats—CRISPR-associated protein systems (Figure 2) allow researchers to precisely edit the genetic code without the insertion of “new genetic material”. Moreover, traits need not be “introduced” to organisms, sometimes the traits that result from gene editing are traits that can occur in the population naturally. And it is not clear why the principle should be restricted to applications that are primarily for human use; if an animal is genetically modified to benefit the ecosystem, there is still good reason to have concern for animals’ welfare. With these minor modifications in mind, I propose that the principle is better formulated as follows:

**Principle for the Conservation of Welfare:** “any animals that are genetically modified through the use of genetic technology, for purposes other than research, should be no worse off, in terms of suffering, than the parent stock was prior to genetic alterations.”

The moral arguments in favor of the principle are fairly straightforward. Though human use of animals is common, most people agree that we ought to avoid causing unnecessary and avoidable suffering. If we have an opportunity to prevent suffering or to avoid the creation of new suffering at little or no cost, and we fail to act on that opportunity, then we have done something wrong. Moreover, many people also consider it to be wrong to use others as a means to an end, particularly when this “use” involves harm. Genetic modifications that harm the resulting animals are naturally seen as using other beings as a means to human ends, which provides additional reasons for adopting the principle.

What reasons might we have for rejecting the principle? One type of argument would result from the claim that “any and all” genetic modifications of animals using genetic technologies are wrong and that the principle is therefore superfluous or, worse, providing cover for immoral practices. However, the potential of genetic modification to improve the environment, human health, and animal welfare provides strong reasons in favor of exploring its potential and the most common arguments for a universal prohibition against genetically modifying animals all fail (Shriver and McConnachie, 2018).

**Figure 3.**
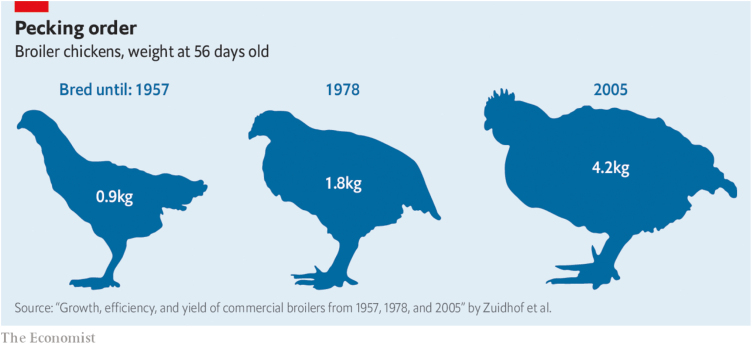
Selective breeding has increased the size of broiler chickens dramatically since 1957. Image source: https://www.economist.com/international/2019/01/19/how-chicken-became-the-rich-worlds-most-popular-meat

A very different type of argument objects to the principle on the grounds that it unfairly holds genetic technologies to a standard that is not seen in other human interactions with animals. For example, selective breeding has been used to produce broiler hens that grow much faster than previous generations (Figure 3), which has resulted in welfare problems (De Jong et al., 2011). The hens produced via selective breeding were not better off, in terms of suffering, in relation to previous generations, and so the practice of using these hens would not meet the standards of the principle. It is true that the principle for the conservation of welfare diverges from standards used for other human interactions with animals; however, the proper response to this divergence is not to weaken the principle, but rather to improve our treatment of animals in other domains. If we believe that it is wrong to cause unnecessary and avoidable suffering, or to harm others as a means to an end, there are good reasons to accept the principle and to use similar principles in connection with other human activities.

Nevertheless, the principle for the conservation of welfare should not be regarded as an absolute prohibition. We can imagine situations where it might need to be violated in order to prevent some catastrophe, or to prevent greater suffering. In fact, the above specification of the principle includes a specific exception for research, which is an acknowledgment that the gains, which may result from gene editing, could potentially outweigh harms that are limited in scope by being restricted to highly controlled research environments. Many moral principles, including “don’t kill” and “don’t steal”, require exceptions for certain circumstances, and this fact need not undermine the general utility of the rule as long as the specific exceptions are made clear. And if we are going to use animals to improve human life, the principle for the conservation of welfare is one necessary step required to ensure that we are acting with appropriate empathy and concern for the welfare of others, as well as acting responsibly in our unique role as caretakers for other animals is followed.

## Win–Wins and Offsetting: Opportunities for Improving Welfare

**Figure 4.**
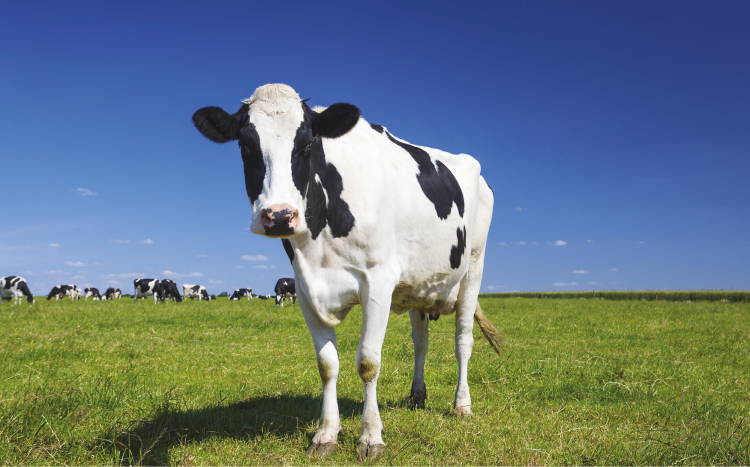
Gene editing can improve animal welfare by eliminating the need for dehorning.

Some proposed genetic modifications are good both for animals and for people. Making animals disease-resistant can improve their welfare and benefit food production systems (Figure 4). Diseases targeted thus far in genetic research include mastitis (Wall et al., 2005; Liu et al., 2014), tuberculosis (Wu et al., 2015; Gao et al., 2017), porcine reproductive and respiratory syndrome (Burkard et al., 2017), and avian influenza virus (Lyall et al., 2011). Similarly, using gene editing to ensure that cows are hornless (Carlson et al., 2016) or that boars do not develop boar taint (Carlson et al., 2014) can be valuable methods of allowing animals to avoid painful procedures such as dehorning and castration. We can imagine other techniques that might eliminate other painful procedures such as tail-docking, debeaking, or branding. These “win–win” modifications are clearly consistent with the principle for the conservation of welfare.

However, some genetic changes to animals performed for the sake of human nutrition, sustainability, or economic benefits might result in decreased welfare for the animals. These changes could be morally justified in a manner consistent with the principle “only” if the changes were offset by additional changes that result in sufficient welfare improvements. In other words, if one modification results in decreased welfare, another modification results in as great or greater improvement of welfare, then the resulting animals will “not” be worse off, in terms of suffering, than the relevant parent stock. Thus, the principle is consistent with, and encourages, genetic modifications specifically designed to improve animal welfare.

Potential proposals that could involve major welfare improvements and hence high potential for offsetting include genetically modifying animals to have reduced capacities to suffer (Shriver, 2009), to be completely insentient, or to have high levels of endogenous opioids. These proposals, if successful, could provide powerful offsets for other types of proposed changes. However, they are at present much more speculative than some of the other proposed modifications above, and serious epistemological challenges would need to be overcome in order to be certain that the welfare of animals was truly improved by such modifications.

## Three Myths Regarding Food Industry Ethics

I hope to have provided a brief though relatively intuitive case for arguing that protecting animal welfare should be a top priority for future genetic modification of animals. However, I will also argue for a stronger claim that this priority should be enshrined in laws and regulations rather than left to individuals and companies. I believe this is both the right thing to do “and” will be required in order to avoid a massive loss of public trust in the current food production system. In order to make this case, I first will address three prominent myths that can lead to the downplaying of ethical concerns in the food industry.

### Myth no. 1: what we eat is purely a “personal choice”

**Figure 5.**
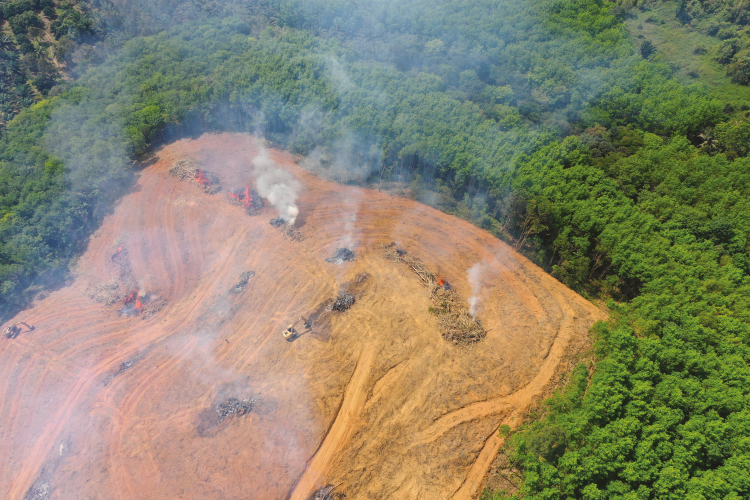
With increased knowledge of food system’s impact on the environment and future generations, food can no longer be regarded exclusively as a “personal choice”.

As Thompson (2016) has noted, there has been a dramatic upsurge in public food ethics as well as philosophical food ethics since around the turn of the twenty-first century. After many critical documentaries and books that focused on the modern food industry, the Amazon rainforests currently on fire (Figure 5), and multiple scientific studies showing the negative effects of current rates of meat consumption, very few people would now argue that the food industry status quo can remain unchanged.

Among the public, and especially younger generations, food has become substantially more political than in past decades. Food choices are wrapped up with concerns about workers, health, land use, pollution, climate change, and animal welfare. A decade ago, many environmental activists would have scoffed at the suggestion that their food choices were relevant for addressing climate change; today, many environmental groups pay close attention to what is served at their events.

Perhaps what is most notable in historical terms is not so much the recent emergence (or reemergence) of the ethical interest in food, but rather the disappearance of food ethics for much of the twentieth century (Thompson, 2015). That century saw radical transformation in how most people obtained their sustenance, with consumers becoming further and further removed from actual food sources. In the context of this great physical and psychological distance, it was easy to see the choices of what one purchased at a supermarket or restaurant as primarily a “personal choice” that was nobody’s business but one’s own. While people were not paying attention, they became less informed of what was happening, and their idyllic view of farming and moral attitudes toward food production became increasingly mismatched with reality. As such, the new “food ethics” movement of the past 20 yr might best be seen as ethics belatedly catching up to the dramatic changes of the last century, and a late realization that the choices we make when purchasing food have many effects on others, and can no longer been seen purely as a “personal choice”.

### Myth no. 2: what is good for business is thereby ethical

The second myth is that as long as food producers are focused on “feeding the world”, they can ignore other types of ethical concerns. While no one doubts that ensuring that people have adequate food and nutrition is an extremely important concern, this noble goal does not negate other concerns. There are many possible systems that can be designed to provide food security, some of which involve radical departures from our current approach, so merely pointing to one food system does not explain why this system should be preferred over others. In regards to livestock in particular, proposed alternative systems for “feeding the world” include shifting to primarily plant-based diets, developing lab-grown meat, and shifting to insect-based diets. All of these approaches have some advantages over the current system, so claiming that we need to continue current livestock practices in order to feed the world is not supported in the absence of further argument and evidence that are responsive to comparisons to other systems.

Moreover, people care about many things and so any particular ethical benefit must be weighed against costs and against other concerns. Looming crises such as those predicted by climate change models suggest that other potential harms resulting from decisions cannot be easily dismissed as obviously “less important” than current food security. Ensuring that everyone is fed is extremely important, and so is preventing environmental catastrophe. The most ethical food system will be one that optimally balances all of the things that we care about.

### Myth no. 3: what is good for business necessarily protects animal welfare

The final myth is that animal welfare always coincides with what is best for business, even in the short term. There is a limited extent to which this is true; animals that don’t survive until slaughter clearly count as economic losses, and Temple Grandin has successfully shown that some types of harm to animals results in lesser quality meat (1995). But believing that welfare and economics “always” coincide with one another requires wishful thinking, with cases like fast-growing broiler chickens providing clear counterexamples. Even believing that welfare and economics “usually” coincide requires adopting a very particular conception of what it means for an animal’s life to go well. One conception of animals “flourishing”, prominent within the veterinary community, is that animals are doing well as long as they are not sick and do not have any obvious health problems; this view fits well with the idea that economics and welfare are closely intertwined. However, as argued effectively by Fraser (2008), this view does not represent the full spectrum of perspectives on what constitutes a good life for animals. The general public sees natural living and the opportunity for positive experiences as necessary components of animal welfare, and simply ignoring these views as the wrong way to think about welfare will do nothing to mollify them.

If anything, the potential for genetic modification increases the potential for divergence between welfare improvements and economic improvements. The ability to make more precise changes in animal phenotypes increases the potential to target very specific traits. With this greater precision, animal welfare will be unlikely to improve substantially unless it is treated as a priority.

## Why Leaving Ethical Choices to Individuals Won’t Work

These three myths, I believe, all implicitly contribute to a view that trusts that ethical values will be reflected in free markets. According to this way of thinking, since food is a personal choice, the state should not be involved in telling or even encouraging people to go for more ethical options. Because good businesses confronts the noble goal of feeding the world (and ensure good welfare in the process), standard market competition will by and large lead to good outcomes. And if consumer values do change to care more about, say, the environment or some new way of thinking about animal welfare, then the markets can adjust to ensure that consumers’ ethical concerns are reflected in their practices.

This approach, I believe, will be a recipe for disaster in regards to gene editing. To see why, consider a hypothetical situation where the majority of food producers act to protect animal welfare but one producer decides to make genetic modifications that decrease the welfare of animals while increasing the economic efficiency of their operation (some examples could include animals that grow much faster or that have substantially more body weight). Over time, everything else being equal, the more economically efficient producer will out-compete those that do not adopt the same practice, and this will eventually push toward widespread use of the decreased welfare modification.

But why not assume that if people genuinely care about welfare, this would prevent the above scenario from occurring? As we have seen in recent history, the dramatic disconnect between the production of food and the purchasing of food means that the reflection of ethical views in business practices is not instantaneous and there can often be a substantial lag, as in years or even decades, in people recognizing disconnects between their values and the food system.

This dynamic, however, is not unique to gene editing, so why think that there are any special risks that would be unique to gene editing? The reason is that gene editing, unlike previous changes to the food system, has the potential to undermine some of the most central positive associations people have with food production. Whereas previous misalignments between moral values and business practices could be viewed by the public as regrettable but ultimately understandable attempts by producers to react to the constraints on their profession posed by the inherent nature of the animals, gene editing involves direct control over the very animals that are being put to use for human benefits. If you make a mistake while trying to design a system responsive to an animal, that’s one thing. But if you design the system “and” the animal, and still make the mistake, there is no excuse.

Consider some of positive associations people have with farming; farmers throughout history been viewed as more closely connected to nature and to the land than city-dwellers, and to be finely attuned experts responsive to the needs of the animals they raise. Moreover, the farming life has been believed to give rise to virtuous traits that represent a unique type of human flourishing. But a situation where consumers learned that genetically modified animals were suffering unnecessarily, years after the practice was implemented that would undercut all of these associations to an unprecedented degree. A farmer failing to prevent suffering in genetically modified animals would not be seen as “connected to nature”, since the animals would, in essence, be laboratory creations. Likewise, the farmer would not be seen as having unique expertise about the animals, since the animals are not direct decedents of a long tradition of husbandry but rather the creation of a different type of specialized knowledge altogether. In short, such a scandal, which I have argued would be very likely to occur with unchecked market forces, would be likely to permanently damage the public’s trust in food production.

Because a market-based ethics approach that left welfare protection in genetic technology up to individuals and companies would be extremely likely to lead to a situation that is both ethically wrong and seriously damaging for trust in the food industry, we should instead prefer an approach that enshrines ethical commitment to welfare in legislation and regulations. This would place firm constrains on the extent to which market considerations could decrease welfare, and would eliminate the “race to the bottom” style market pressure to embrace practices that are bad for welfare. Moreover, including the principle for the conservation of welfare in legislation would communicate clearly that food production is an inherently ethical practice and that food producers view themselves as having commitments to the greater good. This in turn, would help to ensure that the best characteristics of humans, our compassion and empathetic concern for others, are reflected in our future food systems.

## Conclusion

The principle for conservation of welfare states that, “any animals that are genetically modified through the use of genetic technology, for purposes other than research, should be no worse off, in terms of suffering, than the parent stock was prior to genetic alterations.” I have argued that adopting this principle is both the right thing to do and necessary to avoid undermining public trust in the food production system. Moreover, leaving adherence to the principle up to individual actors will not work; the principle needs to be enshrined in the law or regulations in order to avoid market-based pressures that push away from our ethical values.

## About the Author

**Dr Adam Shriver** is a Research Fellow at the Wellcome Centre for Ethics and Humanities and the Oxford Uehiro Centre for Practical Ethics. He received a PhD from the Philosophy-Neuroscience-Psychology Program at Washington University in St. Louis and has worked at the University of Pennsylvania and the University of British Columbia. His research interests include ethics, philosophy of mind, and philosophy of cognitive science.

## References

[CIT0001] Burkard C., Lillico S.G., Reid E., Jackson B., Mileham A.J., Ait-Ali T., Whitelaw C.B., and Archibald A.L. 2017 Precision engineering for PRRSV resistance in pigs: macrophages from genome edited pigs lacking CD163 SRCR5 domain are fully resistant to both PRRSV genotypes while maintaining biological function. PLoS Pathog. 13:e1006206. doi:10.1371/journal.ppat.1006206. 28231264 10.1371/journal.ppat.1006206 PMC5322883

[CIT0002] Carlson D.F., Fahrenkrug S.C., and Lauth X. 2014 U.S. Patent No. US20140123330 A1. Washington: Patent and Trademark Office.

[CIT0090] Carlson, D.F., C.A. Lancto, B. Zang, E.S. Kim, M. Walton, D. Oldeschulte, C. Seabury, T.S. Sonstegard, and S.C. Fahrenkrug. 2016. Production of hornless dairy cattle from genome-edited cell lines. Nat. Biotechnol. 34(5):479–481. doi:10.1038/nbt.3560. 10.1038/nbt.3560 27153274

[CIT0003] De Jong I.C., Perez Moy T., Gunnink H., Van den Heuvel H., Hindle V., Mul M., and Van Reenen. C.G. 2011 Simplifying the welfare quality assessment protocol for broilers, Report 533. Lelystad: Wageningen UR Livestock Research Available from https://edepot.wur.nl/196648

[CIT0004] Fraser D 2008 Understanding animal welfare. Acta Vet. Scand. 50(1):S1. 19049678 10.1186/1751-0147-50-S1-S1 PMC4235121

[CIT0005] Gao Y., Wu H., Wang Y., Liu X., Chen L., Li Q., Cui C., Liu X., Zhang J., and Zhang Y. 2017 Single Cas9 nickase induced generation of NRAMP1 knock-in cattle with reduced off-target effects. Genome Biol. 18(1):13. doi: 10.1186/s13059-016-1144-4 28143571 10.1186/s13059-016-1144-4 PMC5286826

[CIT0006] Grandin T 1995 The economic benefits of proper animal welfare. In: Reciprocal Meat Conference Proceedings; Savoy, IL: American Meat Science Association.

[CIT0007] Liu X., Wang Y., Tian Y., Yu Y., Gao M., Hu G., Su F., Pan S., Luo Y., Guo Z., et al. 2014 Generation of mastitis resistance in cows by targeting human lysozyme gene to β-casein locus using zinc-finger nucleases. Proc. Biol. Sci. 281:20133368. doi:10.1098/rspb.2013.3368 24552841 10.1098/rspb.2013.3368 PMC4027401

[CIT0008] Lyall J., Irvine R.M., Sherman A., McKinley T.J., Núñez A., Purdie A., Outtrim L., Brown I.H., Rolleston-Smith G., Sang H., et al 2011 Suppression of avian influenza transmission in genetically modified chickens. Science 331:223–226. doi:10.1126/science.1198020 21233391 10.1126/science.1198020

[CIT0009] Rollin B 1995 The Frankenstein syndrome: ethical and social issues in the genetic engineering of animals. New York: Cambridge University Press.

[CIT0010] Shriver A 2009 Knocking out pain in livestock: can technology succeed where morality has stalled? Neuroethics 2(3):115–124.

[CIT0011] Shriver A., and McConnachie E. 2018 Genetically modifying livestock for improved welfare: a path forward. J. Agr. Environ. Ethic. 31(2):161–180.

[CIT0012] Thompson P.B 2015 From field to fork: food ethics for everyone. Oxford: Oxford University Press.

[CIT0013] Thompson P.B 2016 The emergence of food ethics. Food Ethics 1(1):61–74.

[CIT0091] Wall, R.J., A.M. Powell, M.J. Paape, D.E. Kerr, D.D. Bannerman, V.G. Pursel, K.D. Wells, N. Talbot, and H.W. Hawk. 2005. Corrigendum: genetically enhanced cows resist intramammary Staphylococcus aureus infection. Nat. Biotechnol. 23(7):897–897. 10.1038/nbt1078 15806099

[CIT0014] Wu H., Wang Y., Zhang Y., Yang M., Lv J., Liu J., and Zhang Y. 2015 TALE nickase-mediated SP110 knockin endows cattle with increased resistance to tuberculosis. Proc. Natl. Acad. Sci. U.S.A. 112:E1530–E1539. doi:10.1073/pnas.1421587112 25733846 10.1073/pnas.1421587112 PMC4386332

